# Supplementary material for: Palm-Sized Wireless Transient Elastography System with Real-Time B-Mode Ultrasound Imaging Guidance: Toward Point-of-Care Liver Fibrosis Assessment
Source: Diagnostics (Basel). 2024 Jan 15;14(2):0. doi: 10.3390/diagnostics14020189 (PMC11154523; doi:10.3390/diagnostics14020189)
Supplement: Supplementary file 1 [file diagnostics-14-00189-s001.zip › diagnostics-2815447-supplementary.pdf]

Supplementary Material

Supplementary Results

Supplementary Figure S1 illustrates the Bland-Altman analyses and Supplementary Figure S2 summarizes the correlation and linearity of liver stiffness of conventional TE-fs against conventional TE-ft, 2D-SWE and Liverscan in the subgroup of 90 patients. As presented in Supplementary Figure S1 (A), conventional TE-fs and Liverscan demonstrated the lowest mean difference of -0.69 kPa (95% limit of agreement: -2.91 to 1.54) among all paired comparisons. Specifically, most difference scores between the techniques were within the limits of agreement and symmetrically distributed around the mean difference. Greater differences in LSMs were observed at the higher mean values which coincided with the result of the 121-patient cohort. Liverscan-derived measurements exceeded conventional TE-derived measurements, with the mean difference of -0.69 kPa indicating minimal overestimation by using Liverscan. This trend was also observed in conventional TE-ft and 2D-SWE with respect to the reference technique of conventional TE-fs (Supplementary Figure (B) and (C)). As assessed by 'goodness-of-fit' in a linear regression model, the linearity of conventional TE-fs against Liverscan (R^2 = 0.617, $P < 0.001$) was nominally higher than that of conventional TE-fs against conventional TE-ft (R^2 = 0.417, $P < 0.001$) and 2D-SWE (R^2= 0.381, $P < 0.001$) (Supplementary Figure S2).

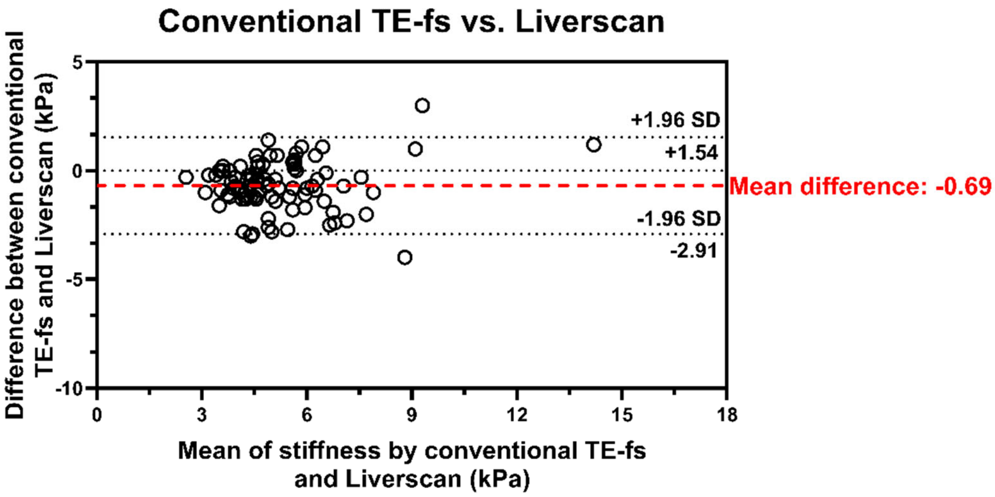

(A)

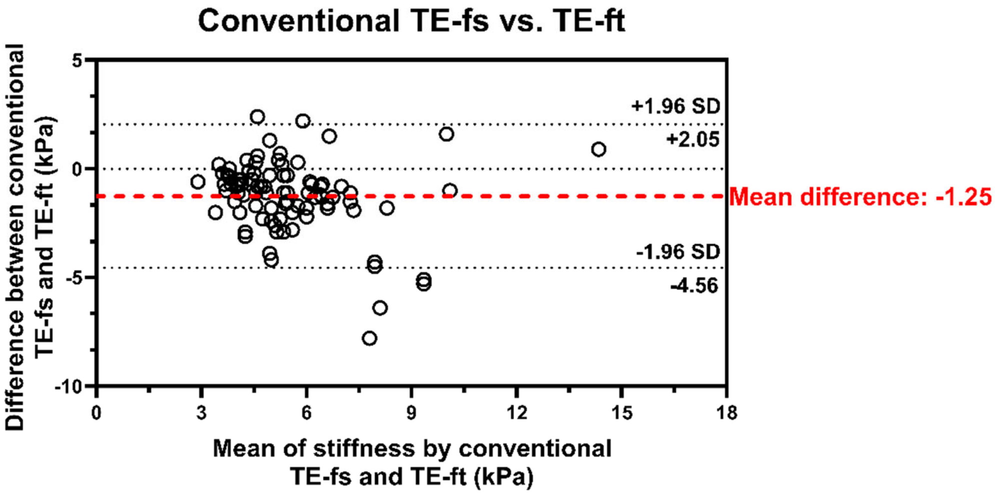

(B)

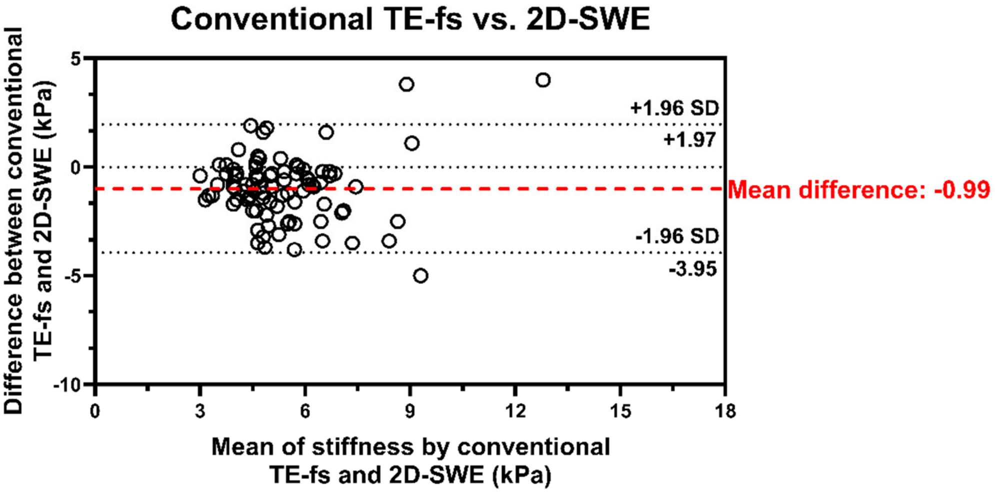

(C)

Figure S1. Bland-Altman plots of liver stiffness for (A) Conventional TE-fs vs. Liverscan; (B) Conventional-fs vs. conventional TE-ft; (C) Conventional TE-fs vs. 2D-SWE.

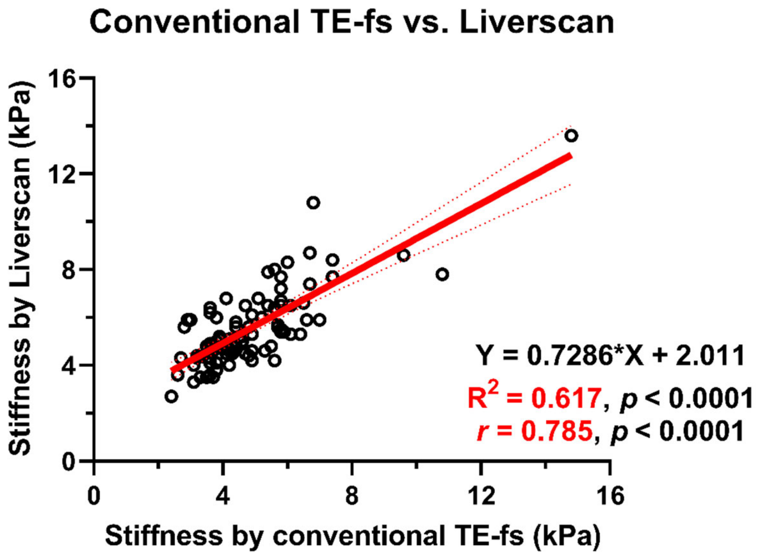
Conventional TE-fs vs. Liverscan
Stiffness by Liverscan (kPa)
Stiffness by conventional TE-fs (kPa)
Y = 0.7286*X + 2.011
R2 = 0.617, p < 0.0001
r = 0.785, p < 0.0001

(A)

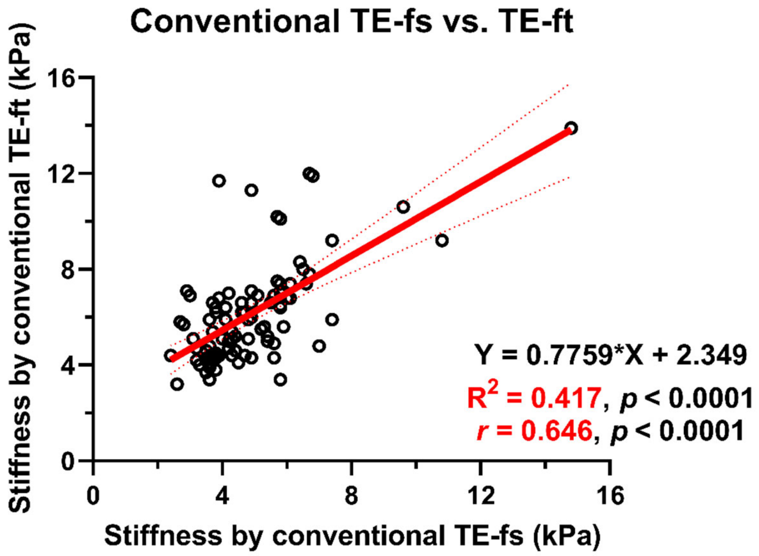
Conventional TE-fs vs. TE-ft
Stiffness by conventional TE-ft (kPa)
Stiffness by conventional TE-fs (kPa)
Y = 0.7759*X + 2.349
R2 = 0.417, p < 0.0001
r = 0.646, p < 0.0001

(B)

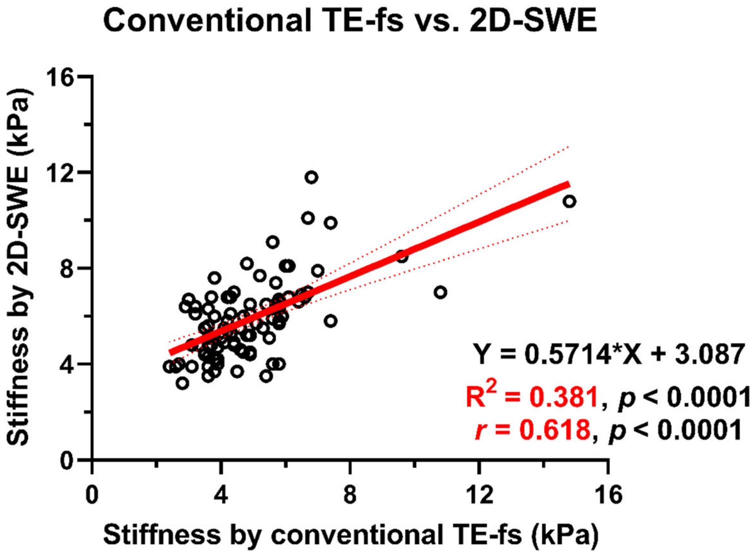

(C)

Figure S2. Scatterplots illustrating linearity and correlation of liver stiffness between (A) Conventional TE-fs vs. Liverscan; (B) Conventional TE-fs vs. conventional-ft; (C) Conventional TE-fs vs. 2D-SWE.
